# Decoding burst swimming performance: a scaling perspective on time-to-fatigue

**DOI:** 10.1098/rsif.2024.0276

**Published:** 2024-10-02

**Authors:** Muhammad Usama Ashraf, Daniel Nyqvist, Claudio Comoglio, Vladimir Nikora, Andrea Marion, Paolo Domenici, Costantino Manes

**Affiliations:** ^1^ Department of Environment, Land and Infrastructure Engineering, Politecnico di Torino, Torino, Italy; ^2^ Department of Engineering, University of Aberdeen, Aberdeen, Scotland, UK; ^3^ Department of Industrial Engineering, Università di Padova, Padova, Italy; ^4^ CNR-IAS, Italian National Research Council, Institute of Anthropic Impacts and Sustainability in the Marine Environment, Oristano, Italy; ^5^ CNR-IBF, Institute of Biophysics, Pisa, Italy

**Keywords:** fish locomotion, swimming performance, burst swimming, fatigue curve, scaling, time-to-fatigue

## Abstract

Fatigue curves quantify fish swimming performance, providing information about the time ($T_{f}$) fish can swim against a steady flow velocity (*U_f_*) before fatiguing. Such curves represent a key tool for many applications in ecological engineering, especially for fish pass design and management. Despite years of research, though, our current ability to model fatigue curves still lacks theoretical foundations and relies primarily on fitting empirical data, as obtained from time-consuming and costly experiments. In the present article, we address this shortcoming by proposing a theoretical analysis that builds upon concepts of fish hydrodynamics to derive scaling laws linking statistical properties of $T_{f}$ to velocities *U_f_*, pertaining to the so-called burst range. Theoretical arguments, in the present study, suggest that the proposed scaling laws may hold true for all fish species and sizes. A new experimental database obtained from over 800 trials and five small-sized Cypriniformes support theoretical predictions satisfactorily and calls for further experiments on more fish species and sizes to confirm their general validity.

## 1. Introduction

Fish swimming performance has drawn a lot of interest in recent decades owing to its importance for fish migration, habitat selection and predator–prey interactions [1–6]. From an applied perspective, fish swimming performance estimates are used extensively in the design of fishways allowing for the passage of fish through dams, weirs, culverts and other anthropogenic barriers [7–9]. Other important applications include the design of sustainable fishing methods [10] and the optimization of practices in the aquaculture industry [11].

Two well-established experimental protocols are commonly used to characterize fish swimming performance: critical velocity and fixed velocity tests [12–14]. Both tests are typically conducted in either a swim chamber or an open channel flume, where fish swim until fatiguing. Fatigue is typically defined as the state of exhaustion where the fish rests at the downstream end of the test section and is not able to swim despite external motivation [15–17]. In the critical velocity test, a fish is forced to swim against a flow velocity *U_f_,* which is progressively increased at fixed time intervals Δ*t*, until the fish fatigues. The velocity and time at which fatigue occurs are then used to compute the critical velocity *U_crit_* [13,18,19]. Fixed velocity tests, on the other hand, consist of repeated swimming trials under a range of fixed velocities. Each trial results in a time-to-fatigue *T_f_*—the time a fish can resist swimming against the defined steady flow velocity (*U_f_*). By repeating trials for different values of *U_f_*, a scatter plot of *T_f_* (dependent variable) versus *U_f_* (independent variable) can be produced. A predetermined model is then fitted to the data to obtain a *fatigue* or *endurance curve* [20]. Fixed velocity tests are more informative than critical velocity tests as they allow for the assessment of fish endurance over a range of flow velocities and associated swimming activity levels [21]. Three of such levels are believed to exist and hereafter are referred to as: sustained, prolonged and burst swimming [12,14,22]. Sustained swimming occurs at velocities whereby fish use primarily red muscles and aerobic processes. Utilizing somatic energy reserves, fish can theoretically maintain sustained swimming indefinitely [12,23]. Prolonged swimming is driven by both red and white muscles, and hence by both aerobic and anaerobic processes. In burst swimming, fish use primarily white muscles and anaerobic processes. Both prolonged and burst swimming are limited by anaerobic energy reserves and therefore subject to exhaustion. By convention, it is assumed that fish can endure prolonged swimming for up to 200 min, while burst swimming is usually associated with $T_{f}≲$ 20 s [9,12]. Actual $T_{f}$ thresholds between burst and prolonged swimming, however, are known to vary with species, size and even amongst similar individuals, so much so burst swimming has been often associated with $T_{f}$ of the order of 1 min or more in the literature [21,24,25].

Our current ability to model fatigue curves is primarily based on empirical mathematical relations between *T_f_* and *U_f_*, which sometimes are supported by dimensional analysis [3,20,21]. Burst and prolonged swimming are commonly associated with fatigue curves following either a log-linear or a power law [3,13,21,23–27]. A theoretical argument in support of either of these laws, however, has never been provided. In their review paper, Katopodis and Gervais [3] collected and analysed a large dataset of fatigue curves, and used a power law model to elucidate relations between *T_f_* and *U_f_* classified by grouping different fish species displaying a similar morphology or swimming kinematics. Nevertheless, the conceptual framework underpinning such relations remains undefined and rooted mostly on empirical grounds. Furthermore, the assessment of Katopodis and Gervais [3] highlighted that a majority of the published fatigue-curve data is limited to prolonged activity. Instead, comparatively little efforts have been made to characterize burst swimming activity [27], despite white muscles constituting the bulk of fish musculature, and burst swimming being key to dictate predator–prey interactions and to overcome velocity barriers [23,28–30], the latter directly relating to the design of fish passage systems [4,9,31].

**Figure 1.**
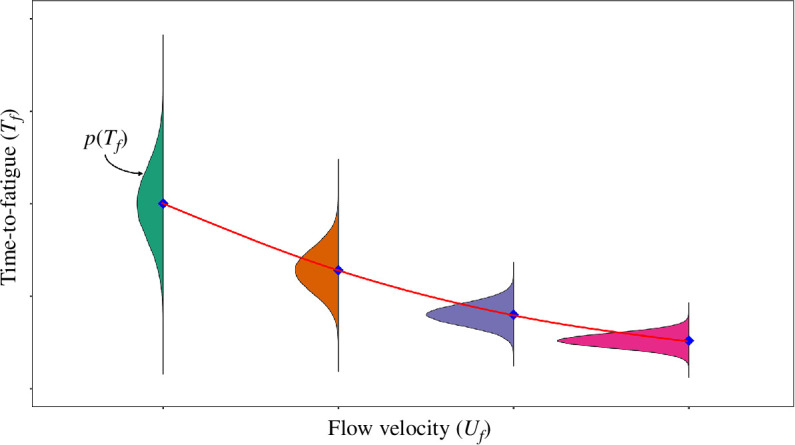
Illustration of the relationship between fish time-to-fatigue $\left(T_{f}\right)$ and flow velocity $\left(U_{f}\right)$. For each test flow velocity value, $p\left(T_{f}\right)$ is the probability density function of related time-to-fatigue. Blue diamonds mark the mean time-to-fatigues at a given test flow velocity. Red curve is the best-fitted line to the entire cloud of data points, referred to as fatigue or endurance curve.

Swimming performance, as estimated in fixed velocity experiments, is characterized by an enormous and unexplored variability where fish species, size and water temperature are often pointed out as key drivers [23,32–36]. Life-stage, sex, experience, health status and nutrition have also been seen to cause variation in fish swimming performance [37–42]. A large variability has also been reported for conspecifics of the same size in response to different fitness and/or motivation [11,35,43]. Therefore, considering a fixed velocity experiment where many fish of the same size and species are tested over a range of flow velocities at constant water temperature, it is reasonable to expect that experimental data will qualitatively spread around mean values of $T_{f}$ (figure 1). For each tested velocity, *T_f_* will display a variability that can be described by a probability function $p\left(T_{f}\right)$. Typically, a fatigue curve is obtained by fitting the entire cloud of experimental data with a predetermined mathematical law (red line in the figure). This, however, only provides information about the general trend of the data but does not provide any clue about $p\left(T_{f}\right)$ and how it varies with *U_f_*.

The aim of this article is to present and validate a theoretical framework allowing for the statistical description of *T_f_* versus *U_f_* data in burst swimming. Towards this end, the following objectives are set:

- to present a theoretical framework based on simple concepts of fish hydrodynamics that can statistically describe the relation between *T_f_* and *U_f_*, showing that $\overset{-}{T_{f}}$ (where $\overset{-}{T_{f}}$ is the mean value of *T_f_*) and central moments of $p\left(T_{f}\right)$ (which indeed help defining $p\left(T_{f}\right)$) vary with *U_f_* following power laws with well constrained and, in principle, universal exponents; and
- to test theoretical predictions in a series of fixed velocity tests using five small-sized Cypriniformes.

## 2. Theoretical framework

In what follows, §2.1 reviews (and refines) the available formulations for fish drag proposed in the literature, while §2.2 draws from §2.1 to derive the sought scaling relations using energetic principles.

### 2.1. Fish drag

The drag force experienced by a fish while swimming can be parameterized as [23]


(2.1) $$F_{D}\sim \rho C_{D}LSU_{r}^{2}$$

where $F_{D}$ is the average drag force, the symbol ‘∼’ stands for *scales as*, ρ is the density of water, *C_D_* is fish drag coefficient, L is total fish length (electronic supplementary material, appendix A, figure A1), S is fish body depth, $U_{r}$ is the relative fish–water velocity, assumed to be identical to the flow velocity in performance tests [21,44]. The estimation of *C_D_* for swimming fish has been a matter of debate for a long time and no shared consensus has been reached [22,45–49]. At high Reynolds numbers (${Re}_{L}=LU_{r}/\nu$, where ν is the kinematic viscosity of water), which are typical for fish swimming at burst velocities, pressure drag is believed to dominate over friction drag [50,51] so that *C_D_* can be considered as a constant and *F_D_* is estimated as


(2.2) $$F_{D}∼\;\rho C_{D}LSU_{r}^{2}\;∼\;\rho LSU_{r}^{2}=\Gamma_{1}U_{r}^{2},$$

where $\Gamma_{1}=\rho LS$ is a function that is herein introduced to lump the effects of parameters pertaining to fish size (i.e. *L* and *S*) and fluid properties (i.e. *ρ*).

The above formulation is, however, questionable as it completely ignores skin friction effects (and hence the dependence of *C_D_* on *Re_L_*), which some researchers argue to be significant, even at high Reynolds numbers [45,52]. While we do not intend to contribute to the debate about the nature of drag in swimming fish, in electronic supplementary material, appendix A, we demonstrate that even when skin friction effects are accounted for and considered the main source of drag, $F_{D}$ can be generally parameterized as


(2.3) $$F_{D}∼\;\Gamma_{j}U_{r}^{\beta },$$

where $\Gamma_{j=1-3}$ ($\Gamma_{2}$ and $\Gamma_{3}$ are defined in electronic supplementary material, appendix A) depend on water properties (ρ and ν) and fish size, shape and tail beat amplitude (i.e. *S*, *L* and *A*), and the scaling exponent β remains well constrained between 1.73 and 2.0. This range of β also accounts for combined effects of skin friction and body undulations on *C*_*D*_.

### 2.2. Energetic considerations and scaling laws

The power (energy per unit time) expended by a fish while swimming can be estimated as $P∼F_{D}U_{r}={𝛤}_{𝑗}{𝑈}_{r}^{𝛽+1}$. Therefore, the total energy spent by an *i*th fish (*E*_*i*_) during a fixed velocity test can be obtained by integrating power (*P*) over time, between zero (i.e. the beginning of the test) and the measured time-to-fatigue (*T*_*fi*_) as


(2.4) $$E_{i}∼\Gamma_{j}\;U_{r}^{\beta +1}T_{fi}.$$

When swimming is dominated by anaerobic processes, as in burst swimming, fatigue occurs when white-fibre muscles burn the available anaerobic reserves down to a critical limit which can be related to $E_{i}$ (through a conversion factor similar to an efficiency coefficient) [12,14,23] and assumed independent of $U_{r}$. Such a critical limit clearly varies extensively among individuals [35,53,54] and cannot be predicted from first principles but it can be described statistically. Towards this end, firstly it should be noted that considering a population of fish of the same species and size and swimming at a constant water temperature, $\Gamma_{j}$ in equation (2.4), depends only on fish size, shape and tail beat amplitude (*L*, *S* and *A*) and water properties (ρ and ν), and hence can be considered constant. Secondly, if averaging is taken over a representative sample of the chosen fish population, the resulting mean energy $\overset{-}{E}$ (overbar indicates averaging), can be also considered as constant. Therefore, averaging both sides of equation (2.4) leads to $\overset{-}{E}\sim U_{r}^{\beta +1}\overset{-}{T_{f}}\approx$ constant, which in turn means that $\overset{-}{T_{f}}$ scales as


(2.5) $$\overset{-}{T_{f}}\;∼\;U_{r}^{-\left(\beta +1\right)}.$$

Using analogous arguments as above, it is possible to derive the scaling of central moments of any order as follows.

First, considering the *i*th fish


(2.6) $$(E_{i}-\;\overset{-}{E})\;∼\;U_{r}^{\beta +1}(T_{fi}-\;\overset{-}{T}).$$

Hence, defining $E_{i}^{\prime }=E_{i}-\;\overset{-}{E}$ and $T_{fi}^{\prime }=T_{fi}-\;\overset{-}{T}$ , equation (2.6) is rewritten as


(2.7) $$E_{i}^{\prime }\;∼\;U_{r}^{\beta +1}\;T_{fi}^{\prime }.$$

By elevating both sides of equation (2.7) to a power *k* and then applying the averaging operator, equation (2.7) transforms into


(2.8) $$\overset{-}{E^{\prime k}}\;∼\;U_{r}^{k\left(\beta +1\right)}\;\overset{-}{T_{f}^{\prime k}}.$$

As per $\overset{-}{E}$, also $\overset{-}{E^{\prime k}}$ can be considered a statistical trait of a fish population that is constant (and therefore independent of $U_{r}$), hence


(2.9) $$\overset{-}{T_{f}^{\prime k}}\;∼\;U_{r}^{-k\left(\beta +1\right)}.$$

Equation (2.9) provides the scaling for central moments $\overset{-}{T_{f}^{\prime k}}$, as sought.

## 3. Material and methods

The study was performed with permission from the Protection of Flora and Fauna Department of the Metropolitan City of Turin (authorization D.D. no. 4457 of 29 October 2020) and the Fauna and Ichthyofauna Technical Office of the Alessandria Province (authorization no. 1570 of 19 January 2023), under the provisions of art. 2 of the national Decree no. 26/2014 (implementation of Dir. 2010/63/EU).

### 3.1. Fish

Experiments were conducted on five freshwater fish species (table 1): Italian riffle dace (*Telestes muticellus*), common minnow (*Phoxinus phoxinus*), European bitterling (*Rhodeus amarus*), North Italian roach (*Leucos aula*) and common bleak (*Alburnus alborella*). These small-sized riverine Cypriniformes were selected because they are all common within their geographic range [55], are classified as least concerned in the IUCN red lists [56], and were expected to display interspecific variation in swimming abilities. Mean fish length ranged between 4.87 and 6.04 cm, with a standard deviation (s.d.) no larger than 0.70 cm for the five fish species (table 1). *Telestes muticellus* and *P. phoxinus* were tested in May–June 2022 and were captured from the Noce stream near Pinerolo, Italy (44°56′17.9″ N 7°23′09.1″ E) on 20 May 2022 and 11 June 2022, respectively, using electrofishing. Fish were transferred to the hatchery facility located in Porte di Pinerolo and were housed in two spring-fed flow-through holding tanks divided into six compartments. *Rhodeus amarus*, *L. aula* and *A. alborella* were tested in January–February 2023. They were captured from the Orba stream in the Province of Alessandria, Italy (44°45′46.7″ N 8°40′15.6″ E) using electrofishing. *Rhodeus amarus* were electrofished on 17 January 2023, whereas *L. aula* and *A. alborella* were caught on 30 January 2023. These fish were brought to the Alessandria Province hatchery in Predosa, Italy and were kept in spring-fed flow-through holding tanks. All fish were allowed to habituate to hatchery conditions for 3–7 days before the experimental trials.

**Table 1.** Summary of experimental data reporting scientific names of the five tested Cypriniformes fish species, the total number of test fish, the number of successful fish trials *n*, test flow velocity *U*_*f*_ values, fish fork length *L*_*f*_, test water temperature *T* and wet fish mass *m*.

				min	max	mean ± s.d.		
scientific names	total test fish	successful trials (*n*)	tested values of *U_f_* (cm s^−1^)	*L_f_* (cm)	*L_f _*(cm)	*L_f_* (cm)	*T* (°C)	*m* (g)
*Telestes muticellus*	202	180	40, 45, 50, 55	4	6.6	4.87 ± 0.46	13.4 ± 0.23	1.56 ± 0.49
*Phoxinus phoxinus*	225	162	50, 55, 60	3.7	6.8	4.90 ± 0.70	16.2 ± 0.31	2.05 ± 0.95
*Rhodeus amarus*	148	90	45, 50, 55	5.4	6.7	6.04 ± 0.34	11.7 ± 0.34	3.13 ± 0.59
*Leucos aula*	160	112	45, 50, 55	4.6	6	5.24 ± 0.36	12.7 ± 0.35	1.85 ± 0.45
*Alburnus alborella*	115	82	45, 50, 55	4.5	6	5.04 ± 0.38	11.4 ± 0.67	1.16 ± 0.31

A HOBO MX-2022 logger was used to record temperature in the holding tanks at 10 min intervals. Temperatures were 13.3 ± 0.4°C (mean ± s.d.) in 2022 and 12.3 ± 0.7°C for the experiments in 2023. All fish were fed commercial aquaria fish pellets (Tetra TabiMin) and were starved at least 24 h before testing to ensure a post-absorptive state [40,57]. Throughout the experiments, fish appeared to be in good health.

### 3.2. Experimental protocol

Experiments were conducted in an open channel recirculating flume with a width of 30 cm and a fixed water depth at any given test flow velocity. The water depth was slightly different for different velocities and ranged from 7 to 9 cm. The swimming arena (flume length) was 60 cm in 2022 and 80 cm in 2023, and delimited by a flow straightener in the upstream direction and a fine-meshed grid in the downstream direction. In a previous study, we demonstrated that such small differences in the length of the swimming arena had no appreciable effect on time-to-fatigue for fish swimming in burst activity level [15]. Trials were recorded from underneath and from the side of the flume using two Sony AX43 handycams with a resolution of 1920 × 1080 pixels at 50 frames per second. A pump allowed water recirculation and the flow rate was manually adjusted using the inverter (DGFIT MT 12) installed with the pump. The flow rate was measured using an AquaTrans^TM^ AT600 flow meter sensor. During trials, water temperature in the system was maintained within a narrow range of 1°C using a chiller unit (TECO TK–2000). The difference between the water temperature in the flume and the holding tanks was kept at less than 1°C to avoid any confounding effects of temperature change on swimming performance [58,59].

All fish were tested individually using a fixed velocity testing protocol. Preliminary tests were conducted on each species to determine which flow velocities could be related to burst swimming [24]. Such tests indicated that flow velocities greater than either 55 cm s^−1^ (European bitterling, common bleak, North Italian roach and Italian riffle dace) or 60 cm s^−1^ (common minnow) resulted in fish simply being unable to swim, hence leading to a large number of unsuccessful trials (a trial where *T_f_* data is not available). Moreover, it was observed that velocities lower than 40 cm s^−1^ (Italian riffle dace), 45 cm s^−1^ (European bitterling, common bleak, North Italian roach) and 50 cm s^−1^ (common minnow) resulted in average times-to-fatigue $\overset{-}{T_{f}}$ significantly exceeding the commonly accepted threshold, in burst swimming, of 20 s. Therefore, fish were tested over a limited range of flow velocities. *Telestes muticellus* were tested at four flow velocities *U_f_* = 40, 45, 50 and 55 cm s^−1^. *Phoxinus phoxinus* were tested at three *U_f_* = 50, 55 and 60 cm s^−1^. The remaining three species, *R. amarus*, *L. aula* and *A. alborella*, were tested at *U_f_* = 45, 50 and 55 cm s^−1^. A single fish was tested per trial at a fixed flow velocity, and no fish was tested more than once. At the beginning of each trial, the fish was habituated for 5 mins at 5 cm s^−1^ [60,61]. The flow rate was then increased manually to achieve the testing flow velocity. Fish were allowed to swim at testing velocity until fatigued. Fatigue was defined as fish resting at the downstream grid and not responding to tapping [16,17,25,62]. A fish was tapped no more than three times during an experimental trial. At the end of the trial, the fish was sedated in clove oil (Aroma Labs, Kalamazoo, MI, USA; approximately 0.2 ml clove oil l^−1^ water), and fork length (cm), mass (g), width (cm) and height (cm) were measured.

### 3.3. Data analysis

In order to test the validity of the scaling relations proposed in §2.2, it was assumed that the mean relative velocity between fish and water *U_r_* could be well approximated by the bulk flow velocity *U_f_* [21]. Experimental data were then used to test the validity of the scaling laws for time-to-fatigue mean ($\overset{-}{T_{f}}$; equation (2.5)) and variance ($\overset{-}{T_{f}^{\prime 2}}$; equation (2.9)). The test was limited to the second-order central moment (*k* = 2), since the estimation of higher orders would have required an enormous amount of data, not available from the above experimental protocol (electronic supplementary material, appendix D).

As outlined in §2.2, statistical properties of $T_{f}$ must be obtained from data pertaining to a population of fish from the same species, having the same size, and swimming at constant temperature. For all fish species, the experimental data were well in line with the constant water temperature requirement (in all trials the water temperature varied over a narrow range of maximum ± 1°C). However, variations in fish size were significant. For example, the fork length *L_f_* (*L_f_* is taken as a proxy for fish size, see electronic supplementary material, appendix C showing allometric relations) varied within the range ± 10.3–38.77%. Hence, following Katopodis and Gervais [3], data were reorganized in subsamples where variations in *L_f_* never exceeded ± 10% (electronic supplementary material, appendix B presents detailed explanation on subsampling procedure).

For all fish species and for each subsampled group separately (electronic supplementary material, appendix B), time-to-fatigue mean ($\overset{-}{T_{f}}$) and variance ($\overset{-}{T_{f}^{\prime \text{2}}}$) were calculated for each tested flow velocity *U*_*f*_. Linear regression was then carried out on log-transformed values of $\overset{-}{T_{f}}$ versus *U*_*f*_, and $\overset{-}{T_{f}^{\prime \text{2}}}$ versus *U*_*f*_ to empirically estimate the exponent *β* in the proposed scaling relations (equations (2.5) and (2.9) with *k* = 2). Results from the regression analysis were deemed acceptable if the null hypothesis of zero slope could be rejected with a 5% significance level (i.e. *p*‐value < 0.05) using Fisher’s test, otherwise, they were discarded.

Since time-to-fatigue data exhibit widespread variability [17,20,63], estimates of $\overset{-}{T_{f}}$ and $\overset{-}{T_{f}^{\prime \text{2}}}$ might be subjected to significant errors unless many data points are available. Moreover, from a statistical standpoint, linear regression in logarithmic coordinates improves when performed over a wide range of velocities in log scale, namely for large values of $ln\left(U_{M}/U_{m}\right)$*,* where $U_{M}$ and $U_{m}$ are the maximum and minimum tested velocities. Small number of data points and low values of $ln\left(U_{M}/U_{m}\right)$ may lead to poor estimates of the scaling exponent (*β*), even if the regression analysis results in high values of $R^{2}$ and *p*-values < 0.05. Therefore, a reliability index (*ReI*) was defined and used to compare the reliability of *β* estimates. Following an approach similar to Jerde *et al*. [64], the *ReI* is defined as


(3.1) $$ReI=p\;ln\left(\frac{U_{M}}{U_{m}}\right),$$

where *p* is the total number of individual data points used for the regression analysis. Equation (3.1) serves as a useful metric to assess the reliability of *β* estimates obtained from datasets of varying quality, regarding the number of samples and range of test flow velocities. Since *ReI* is essentially an index that quantifies the confidence that can be put in the regression of each curve, it is expected that the higher the *ReI* the more likely *β* should fall into the theoretically predicted range.

For all fish species, the distribution of time-to-fatigue data $p\left(T_{f}\right)$ at all test flow velocities was estimated using kernel density estimation (KDE), a non-parametric method to estimate the probability density function (PDF). This was performed to explore whether a working model for $p\left(T_{f}\right)$ could be identified from the available data.

All statistical analyses were run using R version 4.2.2 [65]. Package *dplyr* was used for data management [66], package *ggplot2* was used for plotting [67], package *boot* was used for bootstrap resampling procedure, and package *confintr* was used to calculate the confidence intervals [68].

## 4. Experimental results

A total of 850 fish were tested for the five fish species. Among them, 626 fish (74%) resulted in successful trials where time-to-fatigue was recorded (table 1).

**Figure 2.**
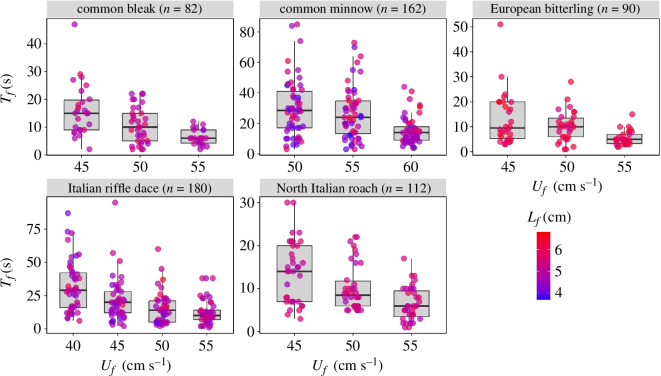
Box plot of all experimentally collected time-to-fatigue (*T*_*f*_) data, without subsampling, for all five fish species superimposed with jitter plot with varying colour intensity based on fish fork length (*L*_*f*_*)*. The solid horizontal black line inside the boxplot marks the median *T*_*f*_ against a test flow velocity *U*_*f*_. The bounding box defines the interquartile range (IQR), containing 50% of time-to-fatigue data. The whiskers mark Q1 – 1.5*IQR (bottom end) and Q3 + 1.5*IQR (top end), where Q1 and Q3 are the 25th and 75th percentiles, respectively.

For all the five fish species $T_{f}$ values show a very large variability at all test flow velocities (figure 2). Mean values of $T_{f}$ are below 20 s except for common minnow and Italian riffle dace as recorded for $U_{f}=40$ and 50 cm s^−1^, respectively (figure 2). Even in these cases, mean values of $T_{f}$ never exceeded 32 s. This means that the great majority of fish were probably tested at burst swimming velocities or very close to. Note that individual values of $T_{f}$ can be instead very large (in some cases exceeding 80 s), meaning that some trials may have occurred under (partly) aerobic swimming conditions.

**Figure 3.**
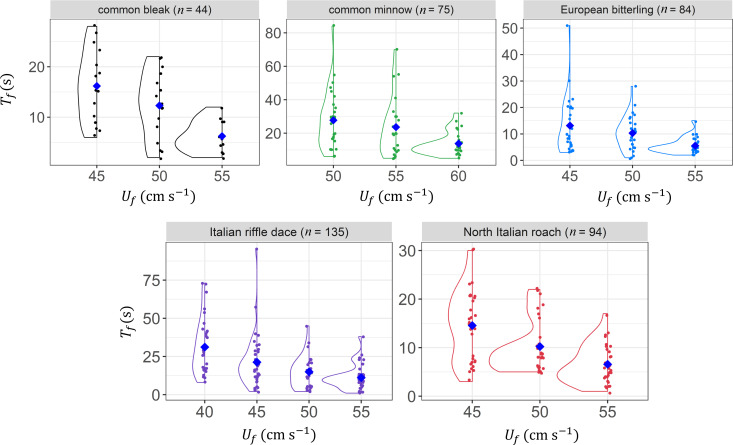
KDE of time-to-fatigue (*T*_*f*_) against flow velocity *U*_*f*_ for the best fit subsampled group (with the highest reliability index value) for the five fish species. Blue diamonds mark the mean time-to-fatigue $\left(\overset{-}{T_{f}}\right)$ at each tested flow velocity.

Despite the large number of tests that were carried out, the KDE of time-to-fatigue data for the subsampled groups does not follow a consistent shape, showing in some cases multimodal while in others unimodal distributions, hence making the identification of a working model for $p\left(T_{f}\right)$ rather difficult. Nonetheless, it is noteworthy that the variability in *T_f_* diminishes with increasing *U_f_* , as theoretically predicted (equation (2.9), k=2). This can be appreciated from figure 3, which shows the KDE related to subsampled groups characterized by the highest *ReI*. Similar conclusions can be drawn from results obtained analysing other subsampled datasets with lower *ReI*.

**Figure 4.**
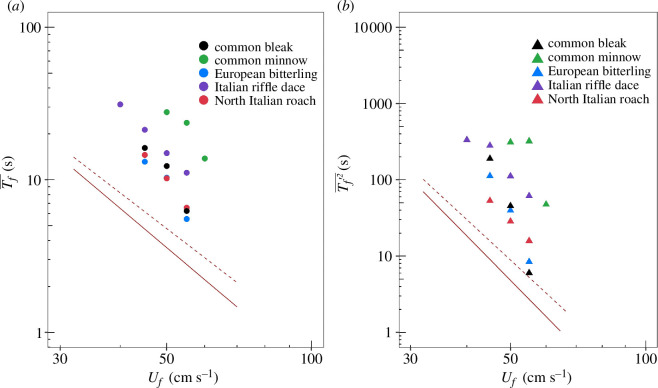
Data for time-to-fatigue mean $\overset{-}{T_{f}}$ (panel (*a*)) and variance $\overset{-}{T_{f}^{\prime \text{2}}}$ (panel (*b*)) versus flow velocity *U*_*f*_ for the subsampled group with the highest reliability index (*ReI*) value. Different colours correspond to different fish species as specified in the legend. Dashed and solid grey lines have slope values calculated from the lower and upper limit of *β* i.e. 1.73 and 2.0, respectively, and are plotted as a benchmark for slope comparison with experimental data (note: the two lines are not empirically fitted fatigue curves). In plot (*a*), the dashed grey line has a slope of − (*β*+1) = −2.73, whereas the solid grey line has a slope of −3. Similarly, in plot (*b*), the dashed grey line has a slope of −2 (*β* + 1) = −5.46, whereas the solid grey line has a slope of −6.

When plotted in logarithmic coordinates, data pertaining to $\overset{-}{T_{f}}$ versus *U_f_* and $\overset{-}{T_{f}^{\prime \text{2}}}$ versus *U_f_* follow straight lines with slopes (which represent the exponent of the power law in linear coordinates) that are in good agreement with theoretical predictions. This is confirmed by figure 4, which reports, as an example, results taken from the subsampled group with the highest *ReI*.

**Figure 5.**
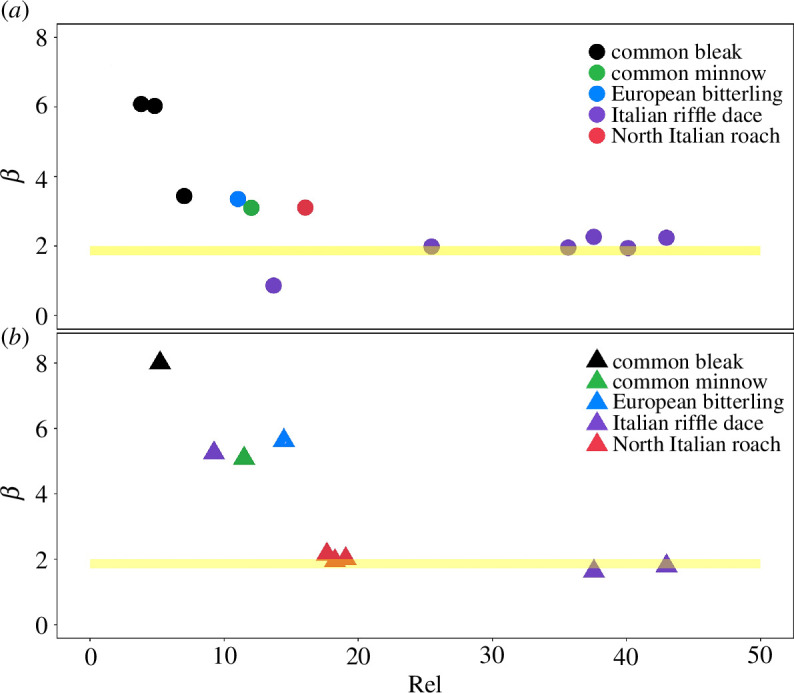
Empirical estimates of scaling exponent *β* obtained from the linear regression analysis between (*a*) ln ($\overset{-}{T_{f}}$) and ln (*U*_*f*_) and (*b*) ln ($\overset{-}{T_{f}^{\prime \text{2}}}$) and ln (*U*_*f*_) plotted against the *ReI*, as defined in equation (3.1). The yellow band is the theoretically predicted range of the scaling exponent *β*, i.e. [1−73−2.0]. Each different colour corresponds to a different fish species as specified in the legend. The repeated data points with the same colour indicate the *β* values obtained from the fitting of more than one subsampled group related to one single fish species.

A more comprehensive view of the results is provided by figure 5, which reports the estimates of the scaling exponent (*β*) obtained from the linear regression analysis of ln $\left(\overset{-}{T_{f}}\right)$ versus ln (*U_f_*) (figure 5*a*) and ln $(\overset{-}{T_{f}^{\prime \text{2}})}$ versus ln (*U_f_*) (figure 5*b*) for all fish species and subsampled groups. For all subsampled groups, there was no effect of fish length on empirical data fitting. The majority of subsampled groups showed a non-significant relationship between time-to-fatigue and flow velocity, probably due to insufficient data, and were consequently omitted from the results (electronic supplementary material, appendix B). For subsamples with significant regression outcomes, results indicate that empirical estimates of *β* closely match the theoretically predicted range (yellow band in figure 5) for Italian riffle dace (figure 5*a*) and both Italian riffle dace and North Italian roach (figure 5*b*), respectively. Interestingly, data points pertaining to low values of *ReI* generally display a larger mismatch with theoretical predictions, and overall, such a mismatch gradually diminishes with increasing values of *ReI*.

## 5. Discussion

This article presents a theoretical approach that predicts scaling laws linking statistical properties of time-to-fatigue $T_{f}$ to the mean relative velocity between water and fish, here estimated as *U_f_*. In particular, theory predicts that $\overset{-}{T_{f}}\sim U_{r}^{-(\beta +1)}$ and $\overset{-}{T_{f}^{`k}}\sim U_{r}^{-k(\beta +1)}$*,* and arguments based upon fish-drag hydrodynamics indicate that the scaling exponent β is constrained between 1.73 and 2.00; the overbar-symbol refers to an averaging operator associated with a population of fish of the same species and size, and swimming in water at a given temperature and velocity *U_f_*; the prime symbol identifies variations of $T_{f}$ around the mean $\overset{-}{T_{f}}$, while *k* is the moment order and can be any integer greater than one. Dedicated fixed velocity experiments were conducted on five Cypriniformes species, and time-to-fatigue data were used to test the proposed scaling relations for time-to-fatigue mean $\overset{-}{T_{f}}$ and variance $\overset{-}{T_{f}^{\prime \text{2}}}$, by comparing theoretically predicted values of β with those estimated from empirical data. Data indicate that empirical estimates of the scaling exponent β agree well with theoretical predictions for Italian riffle dace (as estimated from $\overset{-}{T_{f}}$ data) or Italian riffle dace and North Italian roach (as estimated from $\overset{-}{T_{f}^{\prime \text{2}}}$ data). Overall, deviations from theoretical predictions tend to reduce noticeably with increasing the *ReI* (figure 5). This encouragingly supports the validity of the proposed scaling laws; however, it is also true that the trend is only clearly noticeable for Italian riffle dace, for which data span a large range of *ReI*. Since data for the other four species do not cover the same span (in some cases only one data point is available), there is also a possibility that the extent of the deviations may be species-specific.

It is noteworthy to observe that, at low values of *ReI*, deviations are not uniformly distributed around the theoretically predicted range but biased towards higher values (i.e. mostly larger than 2). This could be explained as follows: the proposed scaling laws apply to fish swimming in the burst range, i.e. in purely anaerobic conditions but, as mentioned in §4, it is possible that some fish employed both anaerobic and aerobic processes during the trials. Clearly, the probability that individual fish swim using aerobic process reduces with increasing *U*_*f*_, meaning that *T*_*f*_ outliers (i.e. large values of *T*_*f*_ caused by aerobic swimming) affect estimates of $\overset{-}{T_{f}}$ and $\overset{-}{T_{f}^{\prime \text{2}}}$ more in the lower range of *U*_*f*_ than in the higher. Since these outliers contribute to increase both $\overset{-}{T_{f}}$ and $\overset{-}{T_{f}^{\prime \text{2}}}$, they also contribute to enhance the steepness of $\overset{-}{T_{f}}$ and $\overset{-}{T_{f}^{\prime \text{2}}}$ versus *U*_*f*_ curves, and hence the values of β estimated from the regression analysis.

The theoretical results reported here can have important practical implications. First, they offer advantages for experimental research aiming at quantifying fatigue in fish. In this respect, note that equations (2.5) and (2.9) can be rewritten, in more general form as


(5.1) $$\overset{-}{T_{f}}=\alpha_{1}\;U_{r}^{-\left(\beta +1\right)},$$

(5.2) $$\overset{-}{T_{f}^{\prime k}}\;=\alpha_{k}\;U_{r}^{-k\left(\beta +1\right)},$$

where $\alpha_{1}$ and $\alpha_{k}$ are scaling functions which depend mainly on fish species, size and water temperature, and β, as discussed, is a well-constrained parameter dictated by theory. This means that, in future studies, it will be possible to explore the statistical behaviour of $T_{f}$ at only one velocity *U_f_* to retrieve the scaling functions $\alpha_{1}$ and $\alpha_{k}$, hence, significantly reducing experimental efforts devoted to the investigation of endurance in burst swimming. Given the enormous biodiversity reported for fish worldwide and the overwhelming variability in swimming performance associated to it, this result is particularly relevant. Second, equation (5.1) represents a useful tool for fishways’ design and management as it allows to derive the maximum distance a fish can swim before becoming fatigued [3,9,20,24]. Recalling the work by Castro-Santos [24] and Katopodis [9], the maximum distance a fish can swim before fatiguing can be defined as $D_{s}=U_{g}\overset{-}{T_{f}}=\left(U_{r}-U_{f}\right)\overset{-}{T_{f}}$, where $U_{g}=U_{r}-U_{f}$ is the fish ground speed. Using equation (5.1) for $\overset{-}{T_{f}}$ leads to a function $D_{s}(U_{r})$ that displays a maximum $D_{smax}$ at an optimal relative velocity $U_{ropt}=U_{f}\left(1+\frac{1}{\beta }\right)$ and hence an optimal ground speed


(5.3) $$U_{gopt}=\frac{U_{f}}{\beta }.$$

Equation (5.3) indicates that, in the burst swimming range, the maximum distance that a fish can travel is reached at swimming ground speeds of about half of the water flow velocity (recall β≈2). This is clearly true only if fish are fit enough to reach such velocity. If not, the maximum distance is reached at the maximum ground speed they are capable to swim at.

At the optimal velocity, the maximum swimming distance can be estimated as

(5.4) $$D_{smax}=\frac{\alpha }{\beta }U_{f}^{-\beta }{\left(1+\frac{1}{\beta }\right)}^{-\beta -1},$$

which represents a very important design parameter being the maximum allowed length for a fish passage system [9,21,24].

Finally, we propose that equation (5.4) might offer some biomimicry-inspired insights for the control of underwater robotics, which are now being used for a plethora of applications [69]. Analogously to a fish swimming anaerobically, an underwater robot stops moving (i.e. reaches fatigue) when running out of the energy provided by a battery. Since the theoretical analysis presented in §§2.1 and 2.2 is applicable to any fully submerged solid body that is self-propelled by limited energy resources, equation (5.4), which is a direct consequence of this analysis, indicates that an underwater robot can maximize cruising distances when swimming at ground speeds that are about half of the opposing fluid velocity. Note that underwater robots cannot be charged during operations; therefore, these insights offer a simple strategy to optimize energy consumption in opposing moving waters, as often required in freshwater and marine environments [70].

In conclusion, the main outcome of the present article is a set of theoretically derived scaling laws linking statistical properties of *T_f_* to *U_r_* for burst-swimming fish. These laws are relevant for applications in fishways’ design and to develop bioinspired underwater-robot control. Results from a new and large experimental dataset of five fish species support theoretical predictions, while calling for more experiments from a wider range of fish species and sizes to be carried out, to further establish the general applicability of the proposed scaling laws.

## Data Availability

The data and code are available in the Zenodo digital repository [71]. Supplementary material is available online [72].

## References

[1] Castro-Santos T. 2002 Swimming performance of upstream migrant fishes: new methods, new perspectives. Dissertation, University of Massachusetts, Amherst, MA. See https://scholarworks.umass.edu/dissertations/AAI3056208/.

[2] Domenici P, Blake R. 1997 The kinematics and performance of fish fast-start swimming. J. Exp. Biol. **200**, 1165–1178. (10.1242/jeb.200.8.1165) 9319004

[3] Katopodis C, Gervais R. 2012 Ecohydraulic analysis of fish fatigue data. River Res. Apps. **28**, 444–456. (10.1002/rra.1566)

[4] Peake S, McKinley RS, Scruton DA. 1997 Swimming performance of various freshwater Newfoundland salmonids relative to habitat selection and fishway design. J. Fish Biol. **51**, 710–723. (10.1111/j.1095-8649.1997.tb01993.x)

[5] Tudorache C, Viaene P, Blust R, Vereecken H, De Boeck G. 2008 A comparison of swimming capacity and energy use in seven European freshwater fish species. Ecol. Freshw. Fish **17**, 284–291. (10.1111/j.1600-0633.2007.00280.x)

[6] Watson J, Goodrich H, Cramp R, Gordos M, Yan Y, Ward P, Franklin C. 2019 Swimming performance traits of twenty-one Australian fish species: a fish passage management tool for use in modified freshwater systems. bioRxiv. (10.1101/861898)

[7] Barbarossa V, Schmitt RJP, Huijbregts MAJ, Zarfl C, King H, Schipper AM. 2020 Impacts of current and future large dams on the geographic range connectivity of freshwater fish worldwide. Proc. Natl Acad. Sci. USA **117**, 3648–3655. (10.1073/pnas.1912776117) 32015125 PMC7035475

[8] Belletti B *et al*. 2020 More than one million barriers fragment Europe’s rivers. Nature **588**, 436–441. (10.1038/s41586-020-3005-2) 33328667

[9] Katopodis C. 1992 Introduction to fishway design. Manitoba, Canada: Freshwater Institute, Central and Arctic Region Department of Fisheries and Oceans.

[10] Castro-Santos T, Goerig E, He P, Lauder GV. 2022 Applied aspects of locomotion and biomechanics. In Fish physiology, pp. 91–140. Cambridge, MA: Academic Press. (10.1016/bs.fp.2022.04.003)

[11] Hvas M, Folkedal O, Oppedal F. 2021 Fish welfare in offshore salmon aquaculture. Rev. Aquacult. **13**, 836–852. (10.1111/raq.12501)

[12] Beamish FWH. 1978 Fish physiology, (eds WS Hoar, DJ Randall), vol. 7, 1st edn. London, UK: Academic Press.

[13] Brett JR. 1964 The respiratory metabolism and swimming performance of young sockeye salmon. J. Fish. Res. Bd. Can. **21**, 1183–1226. (10.1139/f64-103)

[14] Hammer C. 1995 Fatigue and exercise tests with fish. Comp. Biochem. Physiol. A Physiol. **112**, 1–20. (10.1016/0300-9629(95)00060-K)

[15] Ashraf MU *et al*. 2024 Fish swimming performance: effect of flume length and different fatigue definitions. In Advances in hydraulic research [internet] (eds MB Kalinowska, MM Mrokowska, PM Rowiński), pp. 1–11. Cham, Switzerland: Springer Nature Switzerland. (10.1007/978-3-031-56093-4_1). See https://link.springer.com/10.1007/978-3-031-56093-4_1.

[16] Tudorache C, O’Keefe RA, Benfey TJ. 2010 Flume length and post-exercise impingement affect anaerobic metabolism in brook charr Salvelinus fontinalis. J. Fish Biol. **76**, 729–733. (10.1111/j.1095-8649.2009.02513.x) 20666910

[17] Aedo JR, Otto KR, Rader RB, Hotchkiss RH, Belk MC. 2021 Size matters, but species do not: no evidence for species-specific swimming performance in co-occurring great basin stream fishes. Water **13**, 2570. (10.3390/w13182570)

[18] Farrell AP, Steffensen JF. 1987 An analysis of the energetic cost of the branchial and cardiac pumps during sustained swimming in trout. Fish Physiol. Biochem. **4**, 73–79. (10.1007/BF02044316) 24226146

[19] Gregory TR, Wood CM. 1999 The effects of chronic plasma cortisol elevation on the feeding behaviour, growth, competitive ability, and swimming performance of juvenile rainbow trout. Physiol. Biochem. Zool. **72**, 286–295. (10.1086/316673) 10222323

[20] Katopodis C, Gervais R. 2016 Fish swimming performance database and analyses. Canadian Science Advisory Secretariat (CSAS) Research Document 2016/002.

[21] Nikora VI, Aberle J, Biggs BJF, Jowett IG, Sykes JRE. 2003 Effects of fish size, time-to-fatigue and turbulence on swimming performance: a case study of Galaxias maculatus: swimming performance of inanga. J. Fish Biol. **63**, 1365–1382. (10.1111/j.1095-8649.2003.00241.x)

[22] Webb P. 1975 Hydrodynamics and energetics of fish propulsion. Bullettin of the fisheries Research Board of Canada **190**, 1–156.

[23] Videler JJ. 1993 Fish swimming. Dordrecht, The Netherlands: Springer. See http://link.springer.com/10.1007/978-94-011-1580-3.

[24] Castro-Santos T. 2005 Optimal swim speeds for traversing velocity barriers: an analysis of volitional high-speed swimming behavior of migratory fishes. J. Exp. Biol. **208**, 421–432. (10.1242/jeb.01380) 15671330

[25] Videler JJ, Wardle CS. 1991 Fish swimming stride by stride: speed limits and endurance. Rev. Fish Biol. Fish. **1**, 23–40. (10.1007/BF00042660)

[26] Castro-Santos T, Sanz-Ronda FJ, Ruiz-Legazpi J. 2013 Breaking the speed limit—comparative sprinting performance of brook trout (Salvelinus fontinalis) and brown trout (Salmo trutta). Can. J. Fish. Aquat. Sci. (ed. B Jonsson), **70**, 280–293. (10.1139/cjfas-2012-0186)

[27] Haro A, Castro-Santos T, Noreika J, Odeh M. 2004 Swimming performance of upstream migrant fishes in open-channel flow: a new approach to predicting passage through velocity barriers. Can. J. Fish. Aquat. Sci. **61**, 1590–1601. (10.1139/f04-093)

[28] Burnett NJ, Hinch SG, Braun DC, Casselman MT, Middleton CT, Wilson SM, Cooke SJ. 2014 Burst swimming in areas of high flow: delayed consequences of anaerobiosis in wild adult sockeye salmon. Physiol. Biochem. Zool. **87**, 587–598. (10.1086/677219) 25244372

[29] Nyqvist D, Schiavon A, Candiotto A, Mozzi G, Eggers F, Comoglio C. 2023 PIT-tagging Italian spined loach (Cobitis bilineata): methodology, survival and behavioural effects. J. Fish Biol. **102**, 575–580. (10.1111/jfb.15289) 36514841

[30] Taylor EB, McPhail JD. 1985 Burst swimming and size-related predation of newly emerged coho salmon Oncorhynchus kisutch. Trans. Am. Fish. Soc. **114**, 546–551. (10.1577/1548-8659(1985)114<546:BSASPO>2.0.CO;2)

[31] Silva AT *et al*. 2018 The future of fish passage science, engineering, and practice. Fish Fish. **19**, 340–362. (10.1111/faf.12258)

[32] Bainbridge R. 1958 The speed of swimming of fish as related to size and to the frequency and amplitude of the tail beat. J. Exp. Biol. **35**, 109–133. (10.1242/jeb.35.1.109)

[33] Bainbridge R. 1960 Speed and stamina in three fish. J. Exp. Biol. **37**, 129–153. (10.1242/jeb.37.1.129)

[34] Hammill E, Wilson RS, Johnston IA. 2004 Sustained swimming performance and muscle structure are altered by thermal acclimation in male mosquitofish. J. Therm. Biol. **29**, 251–257. (10.1016/j.jtherbio.2004.04.002)

[35] Jones PE, Svendsen JC, Börger L, Champneys T, Consuegra S, Jones JAH, Garcia de Leaniz C. 2020 One size does not fit all: inter- and intraspecific variation in the swimming performance of contrasting freshwater fish. Conserv. Physiol. (ed. S Cooke), **8**, coaa126. (10.1093/conphys/coaa126) 33408868 PMC7772615

[36] Wardle CS. 1975 Limit of fish swimming speed. Nature **255**, 725–727. (10.1038/255725a0) 1134569

[37] Beamish FWH, Howlett JC, Medland TE. 1989 Impact of diet on metabolism and swimming performance in juvenile lake trout, Salvelinus namaycush. Can. J. Fish. Aquat. Sci. **46**, 384–388. (10.1139/f89-050)

[38] Jain KE, Birtwell IK, Farrell AP. 1998 Repeat swimming performance of mature sockeye salmon following a brief recovery period: a proposed measure of fish health and water quality. Can. J. Zool. **76**, 1488–1496. (10.1139/z98-079)

[39] Li J, Lin X, Xu Z, Sun J. 2017 Differences in swimming ability and its response to starvation among male and female Gambusia affinis. Biol. Open **6**, 625–632. (10.1242/bio.022822) 28396491 PMC5450316

[40] Penghan LY, Pang X, Fu SJ. 2016 The effects of starvation on fast-start escape and constant acceleration swimming performance in rose bitterling (Rhodeus ocellatus) at two acclimation temperatures. Fish Physiol. Biochem. **42**, 909–918. (10.1007/s10695-015-0184-0) 26684300

[41] Quintella BR, Mateus CS, Costa JL, Domingos I, Almeida PR. 2010 Critical swimming speed of yellow- and silver-phase European eel (Anguilla anguilla, L.): critical swimming speed of yellow- and silver-phase European eel. J. Appl. Ichthyol. **26**, 432–435. (10.1111/j.1439-0426.2010.01457.x)

[42] Reidy SP, Kerr SR, Nelson JA. 2000 Aerobic and anaerobic swimming performance of individual Atlantic cod. J. Exp. Biol. **203**, 347–357. (10.1242/jeb.203.2.347) 10607544

[43] Goerig E, Castro-Santos T. 2017 Is motivation important to brook trout passage through culverts? Can. J. Fish. Aquat. Sci. **74**, 885–893. (10.1139/cjfas-2016-0237)

[44] Plew DR, Nikora VI, Larned ST, Sykes JRE, Cooper GG. 2007 Fish swimming speed variability at constant flow: Galaxias maculatus. N. Z. J. Mar. Freshw. Res. **41**, 185–195. (10.1080/00288330709509907)

[45] Anderson EJ, McGillis WR, Grosenbaugh MA. 2001 The boundary layer of swimming fish. J. Exp. Biol. **204**, 81–102. (10.1242/jeb.204.1.81) 11104713

[46] Lighthill MJ. 1960 Note on the swimming of slender fish. J. Fluid Mech. **9**, 305–317. (10.1017/S0022112060001110)

[47] Lighthill MJ. 1969 Hydromechanics of aquatic animal propulsion. Annu. Rev. Fluid Mech. **1**, 413–446. (10.1146/annurev.fl.01.010169.002213)

[48] Lighthill MJ. 1970 Aquatic animal propulsion of high hydromechanical efficiency. J. Fluid Mech. **44**, 265. (10.1017/S0022112070001830)

[49] Lighthill MJ. 1971 Large-amplitude elongated-body theory of fish locomotion. Proc. R. Soc. Lond. B. **179**, 125–138. (10.1098/rspb.1971.0085)

[50] Gazzola M, Argentina M, Mahadevan L. 2014 Scaling macroscopic aquatic locomotion. Nat. Phys. **10**, 758–761. (10.1038/nphys3078)

[51] Saadat M, Fish FE, Domel AG, Di Santo V, Lauder GV, Haj-Hariri H. 2017 On the rules for aquatic locomotion. Phys. Rev. Fluids **2**, 083102. (10.1103/PhysRevFluids.2.083102)

[52] Eloy C. 2012 Optimal Strouhal number for swimming animals. J. Fluids Struct. **30**, 205–218. (10.1016/j.jfluidstructs.2012.02.008)

[53] Kolok AS. 1992 the swimming performances of individual largemouth bass (Micropterus salmoides) are repeatable. J. Exp. Biol. **170**, 265–270. (10.1242/jeb.170.1.265)

[54] Marras S, Claireaux G, McKenzie DJ, Nelson JA. 2010 Individual variation and repeatability in aerobic and anaerobic swimming performance of European sea bass, Dicentrarchus labrax. J. Exp. Biol. **213**, 26–32. (10.1242/jeb.032136) 20008358

[55] Freyhof J, Kottelat M. 2007 Handbook of European freshwater fishes. Cornol, Switzerland: Publications Kottelat. See https://portals.iucn.org/library/node/9068.

[56] IUCN. 2023 The IUCN Red List of Threatened Species. Version 2023-1. See https://www.iucnredlist.org.

[57] Schneider EV, Hasler CT, Suski CD. 2019 Swimming performance of a freshwater fish during exposure to high carbon dioxide. Environ. Sci. Pollut. Res. **26**, 3447–3454. (10.1007/s11356-018-3849-2) 30515687

[58] Tudorache C, O’Keefe RA, Benfey TJ. 2010 The effect of temperature and ammonia exposure on swimming performance of brook charr (Salvelinus fontinalis). Comp. Biochem. Physiol. A Mol. Integr. Physiol. **156**, 523–528. (10.1016/j.cbpa.2010.04.010) 20433938

[59] Vezza P, Libardoni F, Manes C, Tsuzaki T, Bertoldi W, Kemp PS. 2020 Rethinking swimming performance tests for bottom-dwelling fish: the case of European glass eel (Anguilla anguilla). Sci. Rep. **10**, 16416. (10.1038/s41598-020-72957-w) 33009464 PMC7532191

[60] Ashraf MU, Nyqvist D, Comoglio C, Manes C. 2024 The effect of in-flume habituation time and fish behaviour on estimated swimming performance. J. Ecohydraul. 1–9. (10.1080/24705357.2024.2306411)

[61] Schiavon A, Comoglio C, Candiotto A, Hölker F, Ashraf MU, Nyqvist D. Survival and swimming performance of a small-sized cypriniformes (Telestes muticellus) tagged with passive integrated transponders. J. Limnol. **82**. (10.4081/jlimnol.2023.2129)

[62] Heuer RM, Stieglitz JD, Pasparakis C, Enochs IC, Benetti DD, Grosell M. 2021 The effects of temperature acclimation on swimming performance in the pelagic mahi-mahi (Coryphaena hippurus). Front. Mar. Sci. **8**, 654276. (10.3389/fmars.2021.654276)

[63] Deslauriers D. 2011 Factors influencing swimming performance and behaviour of the shortnose sturgeon (acipenser brevirostrum). MSC thesis, University of New Brunswick, Saint John, Canada.

[64] Jerde CL, Kraskura K, Eliason EJ, Csik SR, Stier AC, Taper ML. 2019 Strong evidence for an intraspecific metabolic scaling coefficient near 0.89 in fish. Front. Physiol. **10**, 1166. (10.3389/fphys.2019.01166) 31616308 PMC6763608

[65] R team. 2022 The R Project for Statistical Computing. Vienna, Austria: R Foundation for Statistical Computing. See https:// www.R-project.org.

[66] Wickham H, François R, Henry L, Müller K, Vaughan D, Posit Software, PBC. 2023 dplyr: A Grammar of Data Manipulation. See https://cran.r-project.org/package=dplyr.

[67] Wickham H *et al*. 2024 Ggplot2: create elegant data visualisations using the grammar of graphics. See https://cran.r-project.org/package=ggplot2.

[68] Mayer M. 2023 Confintr: confidence intervals https://cran.r-project.org/package=confintr

[69] Cui Z, Li L, Wang Y, Zhong Z, Li J. 2023 Review of research and control technology of underwater bionic robots. Intell. Mar. Technol. Syst. **1**, 7. (10.1007/s44295-023-00010-3)

[70] Li X, Yu S. 2023 Comparison of biological swarm intelligence algorithms for AUVs for three-dimensional path planning in ocean currents’ conditions. J. Mar. Sci. Technol. **28**, 832–843. (10.1007/s00773-023-00960-7)

[71] Ashraf MU, Nyqvist D, Comoglio C, Nikora V, Marion A, Domenici P, Manes C. 2024 Time-to-fatigue data for five cypriniformes fish species and R script for data analysis. Zenodo. https://zenodo.org/records/13275592

[72] Ashraf MU, Nyqvist D, Comoglio C, Nikora V, Marion A, Domenici P *et al*. 2024 Data from: Decoding burst swimming performance: a scaling perspective on time-to-fatigue. Figshare. (10.6084/m9.figshare.c.7423801)39353564

